# Supplementary material for: Post-acute sequelae of COVID-19 among hospitalized patients in Estonia: Nationwide matched cohort study
Source: PLoS One. 2022 Nov 23;17(11):e0278057. doi: 10.1371/journal.pone.0278057 (PMC9683565; doi:10.1371/journal.pone.0278057)
Supplement: S1 Table — (RTF) [file pone.0278057.s001.rtf]

Supplementary tables
S 1 Table. Outcomes measured and definitions
Health outcome	Definition: ICD-10 code	
		
hypothyroidism	E03	
type 2 diabetes	E11	
disorder of lipoprotein	E78	
dementia	F01-03, G30, G31.0 or G31.83	
mood disorders	F30-39	
anxiety	F40-48	
substance abuse	F10-19	
insomnia	F51.0 OR G47.0	
hypertension	I 10-16	
ischemic heart diseases	I 20-25	
other forms of heart diseases	I 30-52	
stroke	I 60-64	
chronic lower respiratory diseases	J40-47	
gastritis and duodenitis	K29	
chronic kidney disease	N18, Z99.2	
chronic liver diseases	K70-77	
