# Supplementary material for: Post-acute sequelae of COVID-19 among hospitalized patients in Estonia: Nationwide matched cohort study
Source: PLoS One. 2022 Nov 23;17(11):e0278057. doi: 10.1371/journal.pone.0278057 (PMC9683565; doi:10.1371/journal.pone.0278057)
Supplement: S2 Table — (RTF) [file pone.0278057.s002.rtf]

S2 Table. Hazard ratios (HR) for major incident post-acute COVID-19 sequelae in hospitalised patients and matched general population controls in Estonia 2020-2021

Outcome	crude HR (95%CI)	adjusted HR (95%CI)	p value adjusted	
Death all cause	3.08 (2.69-3.53)	2.57 (2.23-2.96)	<0.0001	
Readmission all cause	2.17 (1.84-2.18)	1.73 (1.58-1.90)	<0.0001	
Chronic lower respiratory disease	4.09 (2.99-5.60)	4.39 (3.09-6.22)	<0.0001	
Other forms of heart disease	3.74 (2.91-4.80)	3.39 (2.58-4.44)	<0.0001	
Dementia	4.56 (2.67-7.43)	4.50 (2.35-8.64)	<0.0001	
Hypertension	3.57 (2.59-4.92)	2.85 (2.03-4.02)	<0.0001	
Chronic kidney disease	3.95 (1.80-8.68)	14.6 (2.69-78.8)	0.002	
Diabetes 2	3.38 (2.33-4.89)	3.17 (2.11-4.75)	<0.0001	
Chronic liver disease	3.38 (1.95-5.83)	4.20 (2.01-8.77)	<0.0001	
Ischemic heart disease	3.08 (2.35-4.02)	3.01 (2.26-4.01)	<0.0001	
Mood disorder	2.37 (1.63-3.44)	2.18 (1.44-3.29)	<0.0001	
Insomnia	2.25 (1.36-3.73)	2.29 (1.29-4.09)	0.005	
Gastritis and duodenitis	2.07 (1.57-2.71)	1.59 (1.18-2.14)	0.002	
Anxiety	1.85 (1.32-2.58)	1.72 (1.19-2.49)	0.004	
Hypothyroidism	1.75 (0.92-3.33)	1.51 (0.73-3.13)	0.27	
Stroke	1.92 (1.36-2.72)	1.50 (1.01-2.23)	0.04	
Disorder of lipoprotein	1.62 (1.09-2.38)	1.48 (0.97-2.25)	0.07	
Substance abuse	1.57 (0.75-3.31)	1.38 (0.58-3.29)	0.47	
