# Supplementary material for: Post-acute sequelae of COVID-19 among hospitalized patients in Estonia: Nationwide matched cohort study
Source: PLoS One. 2022 Nov 23;17(11):e0278057. doi: 10.1371/journal.pone.0278057 (PMC9683565; doi:10.1371/journal.pone.0278057)
Supplement: S3 Table — (RTF) [file pone.0278057.s003.rtf]

S3 Table. Hazard ratios (HR) for major incident post-acute COVID-19 sequelae in patients admitted to intensive care unit and matched general population controls in Estonia 2020-2021

Outcome	crude HR (95%CI)	adjusted HR (95%CI)	p value adjusted	
Death all cause	3.68 (2.86-3.74)	3.38 (2.59-4.40)	<0.0001	
Readmission all cause	2.38 (2.01-2.81)	2.07 (1.74-2.47)	<0.0001	
Chronic lower respiratory disease	4.89 (2.62-9.15)	6.02 (3.02-11.97)	<0.0001	
Other forms of heart disease	5.70 (3.65-8.91)	5.09 (3.17-8.20)	<0.0001	
Dementia	0.91 (0.91-4.31)	0.72 (0.11-4.59)	0.73	
Hypertension	6.34 (2.88-13.94)	6.16 (2.63-14.4)	<0.0001	
Chronic kidney disease	15.2 (1.69-135.9)	934.2 (1.93-451921…)	0.03	
Diabetes 2	4.0 (1.91-8.37)	4.42 (1.82-10.74)	0.001	
Chronic liver disease	1.65 (0.61-4.64)	2.80 (0.71-10.9)	0.14	
Ischemic heart disease	4.57 (2.78-7.53)	4.65 (2.73-7.93)	<0.0001	
Mood disorder	5.02 (2.21-11.38)	7.16 (2.79-18.3)	<0.0001	
Insomnia	3.66 (1.37-9.76)	5.66 (1.72-18.62)	0.004	
Gastritis and duodenitis	3.05 (1.83-5.11)	2.41 (1.36-4.26)	0.002	
Anxiety	1.27 (0.59-2.74)	1.33 (0.58-3.03)	0.67	
Hypothyroidism	2.60 (0.58-11.76)	2.02 (0.36-11.38)	0.43	
Stroke	1.79 (0.89-3.59)	1.61 (0.75-3.42)	0.22	
Disorder of lipoprotein	1.23 (0.54-2.82)	1.27 (0.52-3.11)	0.59	
Substance abuse	3.18 (0.63-16.1)	16.67 (0.70-394.9)	0.08	
