# Supplementary material for: Post-acute sequelae of COVID-19 among hospitalized patients in Estonia: Nationwide matched cohort study
Source: PLoS One. 2022 Nov 23;17(11):e0278057. doi: 10.1371/journal.pone.0278057 (PMC9683565; doi:10.1371/journal.pone.0278057)
Supplement: Attachment — Submitted filename: Response to Reviewers.rtf [file pone.0278057.s004.rtf]

RESPONSE TO REVIEWERS 
General comment:
In addition to the reviewers' comments and suggestions, more clarifications should be written regarding the methods and diagnostic codes (ICD-10) using 1 or 2 codes to confirm the diagnosis of diseases.

Response: Thank you for the comment. We have edited Methods section to clarify the use of ICD-10 codes.


Reviewer #1:
1.	Despite cases and controls were matched by age, sex and CCI, the prevalence of relevant comorbidities was higher in the cohort of patients with COVID-19 (e.g., arterial hypertension, neoplasms, type 2 diabetes, ischemic and non-ischemic heart disease, etc). I wonder whether it is possible to do another approach taking into consideration for matching conditions with a high prevalence (for example, > 5%) in both cohorts.

Response: Thank you for pointing that out.
In the addition to the matching for age, sex, Charlson Comorbidity Index the final Cox survival model was adjusted for comorbidities with prevalence of ≥5%, health care utilization and previous hospitalization that we believe removed confounding if imbalance existed after matching.

2.	In table 2 it is presented the incidence of outcomes among COVID-19 and control patients. Two different measures are presented: person-time and rate (both by 10.000 person-years). Despite I cannot repeat your calculations, rate was higher in COVID-19 patients than in controls (for example, in the case of death from all cause the figures were 1332 and 467, respectively). Conversely, when the same results are presented as person time the figure is higher in controls (1.19 vs. 0.29 for all cause mortality). Can you explain me this discrepancy?
Response: Cases were matched to a random subset of individuals insured by the EHIF who at the date of the first positive SARS-CoV-2 test of the COVID-19 case were alive and had no evidence of COVID-19 using a case to control ratio of 1:4 and that is the reason for person time being 4 times higher for controls (controls 1.19 versus cases 0.29 x 10 000 person years).

Reviewer #2:
1.	Authors wanted to examine post-acute sequelae of Covid-19 disease with a matched cohort study in Estonia. Data came from nationwide healthcare registry. Two patient groups were matched according to age, gender, and CCI score; Covid-19 and others. Although, these three features (age, gender and CCI score) were used for matching, it is obvious that Covid-19 patients had higher rate of pre-existing comorbidities than those of counter parts. So, it is expected that Covid-19 patients could have higher rates of hazard ratios or mortality, and results could be overestimated. Propensity score matching could be more appropriate to reduce these differences in the analysis. If it is not possible to do this test, this limitation should be added to the paper.
Thank you for your comment, we do agree that the propensity score matching would be also another appropriate way to balance exposed groups on the confounding factors, but we believe that matching for for age, sex, Charlson Comorbidity Index and adjusting final Cox survival model for comorbidities with prevalence of ≥5%, health care utilization and previous hospitalization has removed confounding as imbalance existed after matching.

2.	Other concern is that how does this paper give contribution to the literature, as nearly all the population was vaccinated in Europe. Also, we still don't know whether vaccination changed long term disease effects. Or, how long time should these patients will be watched for sequelae? These are topics that should be discussed in the paper.
Our findings are consistent with emerging evidence from early studies suggesting that a subset of people infected with SARS-CoV-2 can experience health problems several months after the acute phase of their infection; however, epidemiological characterisation of such sequelae has been limited. Only a few other studies to date have compared post-COVID risks with a control group in using nationwide data.
The present study helps to contextualise these observations by adding explicit comparison with risks experienced by the general population. In addition we believe that our results can be generalised to those countries with similar population structure with lower vaccination coverage.
We also believe that our results are relevant in terms of future and present pandemics as we note in the manuscript that the post-infectious sequelae have been documented following infection by other viral infections (Ebola, influenza, dengue) and only 63.2% of European population is fully vaccinated ^1^.

1 https://ourworldindata.org/covid-vaccinations?country=OWID_WRL

Ongoing monitoring will be important to investigate whether these patterns persist in the light of new variants and increasing levels of vaccination.
3.	Line 135: “all-cause” rather than “all cause”
Response: corrected
4.	Line 189: It is stated that “At baseline compared to general population cases were more likely to have a comorbid condition (most notably obesity …” Is there any information regarding obesity in the paper?
Response: Thank you for pointing that out. We have now included it in the Tabel 1, we double- checked and it was already included in the final model.
5.	It was used that “age, gender and co-morbidities matched general population.” in a few sentences. Actually, it wasn't a fully matched cohort. It can be used “CCI score” rather than “co-morbidities”
Response: corrected
6.	The results may not provide “robust” estimates for post disease morbidity because the groups were not fully matched. This statement needs revision.

Response: The reviewed statement is now included.
