# Supplementary material for: Post-acute sequelae of COVID-19 among hospitalized patients in Estonia: Nationwide matched cohort study
Source: PLoS One. 2022 Nov 23;17(11):e0278057. doi: 10.1371/journal.pone.0278057 (PMC9683565; doi:10.1371/journal.pone.0278057)
Supplement: Attachment — Submitted filename: Response to Reviewers2.docx [file pone.0278057.s005.docx]

RESPONSE TO REVIEWERS

Reviewer #1:

The limitation that both reviewers have raised about the matching procedure of both cohorts is not fully addressed. Is it possible to match both populations by the baseline comorbidities (for example by a propensity score)?

Response: We are sorry that the matching procedure was not presented clearly. In the analysis presented both populations are matched by the baseline comorbidities burden (Charlson Comorbidity Index).

In our analysis, we matched our cohorts for age, sex, and Charlson Comorbidity Index and adjusted the final Cox survival model for a rich set of covariates to remove confounding as imbalance existed after matching. Adjustment for preexisting comorbidities with a prevalence of ≥5%, health care utilization, and previous hospitalization was made. We believe that this was sufficient to balance the cohorts out. We do agree with the reviewer, that the propensity score matching would be also another appropriate way to balance exposed groups on the confounding factors.

We have addressed this comment by adding an explanation in the Discussion section on page 20 line 359.
